# Protein Tyrosine Phosphatase SHP-2 (*PTPN11*) in Hematopoiesis and Leukemogenesis

**DOI:** 10.1155/2011/195239

**Published:** 2011-06-07

**Authors:** Xia Liu, Cheng-Kui Qu

**Affiliations:** Division of Hematology and Oncology, Department of Medicine, Center for Stem Cell and Regenerative Medicine, Case Comprehensive Cancer Center, Case Western Reserve University, Cleveland, OH 44106, USA

## Abstract

SHP-2 (*PTPN11*), a ubiquitously expressed protein tyrosine phosphatase, is critical for hematopoietic cell development and function owing to its essential role in growth factor/cytokine signaling. More importantly, germline and somatic mutations in this phosphatase are associated with Noonan syndrome, Leopard syndrome, and childhood hematologic malignancies. The molecular mechanisms by which SHP-2 mutations induce these diseases are not fully understood, as the biochemical bases of SHP-2 functions still remain elusive. Further understanding SHP-2 signaling activities and identification of its interacting proteins/substrates will shed light on the pathogenesis of *PTPN11*-associated hematologic malignancies, which, in turn, may lead to novel therapeutics for these diseases.

## 1. Introduction

SHP-2, encoded by *PTPN11*, is a ubiquitously expressed protein tyrosine phosphatase (PTP) that contains two tandem Src homology 2 (SH2) domains, a PTP domain, and a C-terminal tail with tyrosyl phosphorylation sites and a prolyl-rich motif [1–5]. It shares a similar overall structure and high homology with SHP-1, another SH2 domain-containing PTP that is predominantly expressed in hematopoietic cells [6, 7]. However, SHP-2 and SHP-1 play opposing roles in hematopoietic cell function [8–10]. Genetic analyses using the *Xenopus* model have revealed that both the SH2 domains and the PTP domains contribute to their signaling and thus functional specificities [11, 12]. The 2.0 Å X-ray crystal structure of SHP-2 reveals that the protein is self-inhibited by the binding of the N-terminal SH2 (N-SH2) domain to the PTP domain via hydrogen bonds [13, 14]. Upon growth factor/cytokine stimulation, binding of SHP-2 via its SH2 domains to phosphorylated tyrosine residues on growth factor receptors or docking proteins results in disruption of the autoinhibitory interaction, leading to exposure of the catalytic site and enzymatic activation [13, 14]. Remarkably, mutations in *PTPN11* have been identified in several human diseases, such as the developmental disorders Noonan syndrome (NS) [15] and Leopard syndrome (LS) [16, 17], childhood hematologic malignancies [18–20], and sporadic solid tumors [21]. Most of these disease-associated mutations affect N-SH2 or PTP domain residues involved in the basal inhibition, resulting in either “activated mutants” or “inactivated mutants” of SHP-2. Although significant progress has been made in the past years, signaling mechanisms of SHP-2 and the molecular mechanisms by which SHP-2 mutations are associated with human diseases are still not fully understood. Emerging evidence has indicated that SHP-2 may also have important functions in the nucleus and the mitochondria [22–24]. Better understanding the role of SHP-2 in these organelles may provide new insights into the pathogenesis of SHP-2 mutation-associated human diseases. This paper focuses on the physiological and pathological roles of SHP-2 in hematopoietic cell development and leukemogenesis.

## 2. SHP-2 in Cytoplasmic Signal Transduction

 SHP-2 is primarily localized to the cytosol and has been implicated in cell signaling initiated by growth factors, cytokines, and hormones, regulating cell survival/growth and differentiation [8, 25]. In addition, SHP-2 modulates cell adhesion molecules-induced signal transduction and has been found to play an important role in cell migration and motility [26–30]. This phosphatase plays complicated roles in cell signaling processes. It is involved in a variety of cell signaling cascades, such as the RAS-MAP kinase, JAK-STAT, PI3K-AKT, Rho, NF-*κ*B, and NFAT pathways [8, 25]. Furthermore, it acts at multiple sites in individual pathways. SHP-2 appears to function in cell signaling in both catalytic-dependent and -independent manners. While its catalytic activity is important for cell signaling, SHP-2 can also function as an adaptor independent of catalytic activity [31–33]. Despite extensive studies over the past decade, the mechanisms of SHP-2 action are still poorly defined. SHP-2 interacts with a number of cell signaling components, such as growth factor/cytokine receptors, SIRP*α*/SHPS-1, PZR, Grb2, FRS, IRS-1, Gab1, Gab2, p85, STAT5/3/1, and Sprouty [8, 25]. Of these partners, some are the targets of SHP-2 enzymatic activity. However, none of the putative substrates identified to date can fully account for the signaling effects of SHP-2 on the many biological processes with which it has been implicated. PTPs were originally thought to be negative regulators owing to their opposite roles to protein-tyrosine kinases (PTKs). However, SHP-2 is a PTP that plays an overall positive role in many cell-signaling processes, in particular, the RAS-ERK pathway, promoting cell growth and differentiation. The underlying mechanisms remain elusive. In most cases, SHP-2 is considered to regulate an upstream element necessary for RAS activation; yet, SHP-2 functions downstream of, or parallel to, RAS activation have also been demonstrated [34]. The PTP activity of SHP-2 has been shown to be required for full activation of RAS. Three possibilities have been proposed [35]. First, SHP-2 promotes RAS activation by dephosphorylating tyrosine-phosphorylated sites of receptor PTKs or docking proteins that bind p120 RAS-GTPase activating protein (RAS-GAP), a negative regulator of RAS activity; second, SHP-2 potentiates activation of Src family kinases (SFKs) by dephosphorylating Csk (a negative regulator of SFKs) binding protein (Cbp), thereby preventing access of Csk to SFKs. Enhanced activation of SFKs in turn leads to optimal RAS activation; finally, SHP-2 promotes RAS activation by dephosphorylating Sprouty, another negative regulator of RAS. Although in most circumstances, SHP-2 plays a positive role in transducing signals relayed from receptor PTKs, SHP-2 can also function as a negative regulator depending upon stimuli, binding partners, and interacting downstream signaling networks [36]. For example, it negatively regulates JAK/STAT signaling initiated by interferon-*α* and -*γ* [37]. Another prominent example is that SHP-2 negatively regulates gp130 signaling triggered by leukemia inhibitory factor, ciliary neurotrophic factor, and interleukin-6 (IL-6) [38, 39].

## 3. SHP-2 in the Nucleus and the Mitochondria

Emerging evidence shows that SHP-2 is distributed to the nucleus and the mitochondria, where it can also have important functions [22–24]. Prolactin stimulation of mammary cells leads to the nuclear translocation of SHP-2 as a complex with STAT5 which then binds to DNA and regulates transcription of milk protein genes [22]. Also, it has been demonstrated that nuclear SHP-2 dephosphorylates STAT1 and STAT3 at tyrosine and serine residues and thus inhibits their transcriptional activities [40, 41]. Recently, SHP-2 has been found to inhibit nuclear export of telomerase reverse transcriptase (TERT), the catalytic subunit of telomerase, in the nucleus by dephosphorylating Tyrosine^707^ of TERT, thereby enhancing nuclear telomerase activity [42]. Since telomerase plays an important role in maintaining telomere length and shortening of telomeres has been linked to chromosomal instability and cell aging, SHP-2 may thus be involved in the regulation of aging processes. Our previous studies showed that about 30%–40% of SHP-2 is localized to the nucleus in mouse embryonic fibroblasts and that SHP-2 plays an important role in DNA damage-induced cellular responses [23]. SHP-2 promotes DNA damage-induced apoptosis by enhancing the c-Abl kinase-mediated pathway [23]. It is also required for DNA damage-induced translocation of Cdc25C from the nucleus to the cytoplasm [43]. Loss of functional SHP-2 decreases DNA damage-induced apoptosis as well as the cell-cycle checkpoint (G_2_/M) response [23, 43]. Using “rescue” approaches, we have defined that SHP-2 functions in DNA damage-induced c-Abl activation, and thereby apoptosis, in a catalytic-dependent manner, whereas its role in the DNA damage-induced G_2_/M checkpoint does not require catalytic activity [44]. 

SHP-2 is also distributed to the mitochondria, specifically the intercristae/intermembrane space (IMS) [24, 45]. However, the role of SHP-2 in the mitochondria remains unclear. The mitochondrial oxidative phosphorylation (OxPhos) system provides the vast majority of cellular energy and produces reactive oxygen species (ROS). Recently, Lee et al. propose that OxPhos complexes might be direct or indirect targets of SHP-2 [46]. They have found that cytochrome *c* oxidase (CcO) activity and ROS levels are significantly increased, whereas mitochondrial membrane potential and ATP content are decreased in constitutively active SHP-2 mutant cells. CcO subunit II can be phosphorylated by Src kinase which is also localized to the IMS [47, 48]. As it has long been recognized that cytosolic SHP-2 and Src regulate each other, their observations raise the possibility that SHP-2 enhances CcO activity in the mitochondria by activating Src kinase. However, other signaling pathways may also be affected by mitochondrial SHP-2. Identification of mitochondrial substrate(s) and downstream target(s) of SHP-2 will shed light on the molecular mechanisms by which SHP-2 regulates mitochondrial function and mitochondria-dependent cellular activities.

## 4. SHP-2 in Normal Hematopoiesis and the Immune System

Hematopoiesis is the cellular process in which all types of blood cells including erythroid, myeloid, and lymphoid cells are produced from pluripotent hematopoietic stem cells (HSCs). It is tightly controlled by environmental cues, such as cytokines and growth factors. Dysregulation of cytokine/growth factor signaling can result in blood disorders, including hematologic malignancies. SHP-2 is highly expressed in hematopoietic cells. Our previous studies have demonstrated that in contrast to the highly related SHP-1 phosphatase which negatively regulates hematopoietic cell development [49, 50], SHP-2 plays an overall positive role in hematopoiesis [51–53]. N-SH2 deletion generates a loss of function mutation in SHP-2. This mutation severely suppresses the development of erythroid and myeloid progenitors in homozygous mutant embryos [51]. Consistent with this result, neither erythroid nor myeloid progenitors derived from mutant embryonic stem (ES) cells with the N-SH2 deletion mutation in SHP-2 were detectable in the fetal liver or bone marrow of the chimeric animals generated from these ES cells [52]. In addition, SHP-2 is required for lymphopoiesis [53]. In Rag2-deficient blastocyst rescue experiments, differentiation of lymphoid lineages from ES cells with the N-SH2 deletion mutation of SHP-2 was blocked before pro-T- and pro-B-cell stages [53]. These data suggest that SHP-2 is required for the development of all blood cell lineages. Recent studies have demonstrated that SHP-2 plays a critical role in the survival and maintenance of HSCs. Even heterozygous N-SH2 deletion mutation in SHP-2 causes the HSC pool to reside in a less quiescent (G_0_) state. HSC repopulating capacity and self-renewal are markedly reduced by this mutation [54]. Furthermore, depletion of SHP-2 from hematopoietic cells in SHP-2 conditional knockout mice results in rapid loss of HSCs and immature progenitors of all hematopoietic lineages in a gene dosage-dependent and cell-autonomous manner [55].

**Figure 1:**
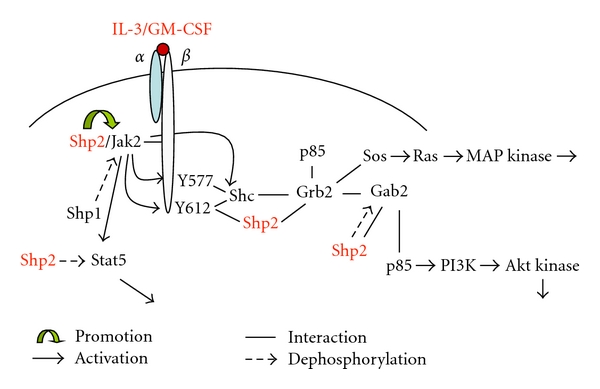
Catalytic-dependent and -independent roles of SHP-2 in IL-3/GM-CSF signaling.

The signaling mechanisms by which SHP-2 regulates hematopoietic cell development and function are not well understood. SHP-2 participates in the signal transduction of many hematopoietic cytokines, such as IL-3, IL-5, IL-6, IL-9, IL-11, EPO, SCF, GM-CSF, M-CSF, and Flt3-ligand [9]. SHP-2 appears to promote hematopoietic cell development by positively regulating critical signaling processes of hematopoietic growth factors/cytokines. We have demonstrated that SHP-2 is required for the signal transduction of IL-3, a cytokine involved in hematopoietic cell survival, proliferation, and differentiation [33]. IL-3-induced activation of JAK/STAT, ERK, and PI3K pathways in hematopoietic cells with N-SH2 deletion mutation of SHP-2 was impaired [33]. However, in catalytically deficient SHP-2 C459S-overexpressing cells, IL-3-induced PI3K activation remained unaltered, while activation of JAK2 and ERK was reduced [33]. These observations suggest that SHP-2 plays multiple roles in IL-3 signal transduction, acting in both catalytic-dependent and -independent manners in the JAK/STAT, ERK, and PI3K pathways (Figure 1). Interestingly, SHP-2 also has a negative effect on hematopoietic cell survival. Overexpression of wild-type SHP-2 in hematopoietic cells compromised their hematopoietic activities and enhanced cytokine deprivation-induced apoptosis by dephosphorylating STAT5 [56]. 

SHP-2 also plays an important role in the regulation of the immune system through its effects on cytokine and inhibitory receptor signaling pathways [57]. In addition to its well-established role in cytokine responses, SHP-2 has been implicated as an important mediator of inhibitory receptor signaling. Inhibitory receptors contain one or more immunoreceptor tyrosine-based inhibitory motifs (ITIMs) within their cytoplasmic domains essential for generation and transduction of inhibitory signals. Tyrosine phosphorylation of the ITIM allows it to bind and activate phosphatases containing SH2 domains. SHP-2 has been found to be recruited to several inhibitory receptors, including the T-lymphocyte-associated antigen 4 (CTLA-4), programmed death-1 (PD-1), B- and T-lymphocyte attenuator (BTLA), killer cell Ig-like receptors (KIRs), CD31 in lymphocytes, and ITIM-containing receptors in granulocytes and platelets [57]. The role of SHP-2 in T cells is still controversial. Frearson and Alexander [58] showed that SHP-2 played a positive role in T cell receptor (TCR) signaling. Expression of catalytically deficient SHP-2 C459S significantly inhibited TCR-induced activation of ERK, but had no effect on TCR-zeta chain tyrosine phosphorylation or TCR-elicited Ca^2+^ transients. However, another study implicates SHP-2 as a negative regulator in TCR signaling [59]. Overexpression of Gab2, a critical downstream substrate/target of SHP-2, in T cells resulted in downregulation of TCR-mediated NFAT activation and IL-2 production [59]. Gab2 mutants lacking SHP-2-binding sites abrogated the inhibitory activity of Gab2, but its inhibitory function was restored by fusing to active SHP-2 as a chimeric protein, suggesting that the inhibitory function of Gab2 is largely dependent on SHP-2 [59]. Additionally, SHP-2 might be involved in the downregulation of T-cell adhesion processes [60]. TCR-induced ROS production selectively inhibits SHP-2-mediated dephosphorylation of Vav1 and ADAP associated with SLP-76, which limits TCR-induced adhesion and integrin clustering [60]. In consistent, overexpression of SHP-2 C459S enhanced TCR-induced LFA-1 clustering and the adhesion of Jurkat T cells to fibronectin [60].

Recent mouse studies have provided new insights into the role of SHP-2 in immune responses. Transgenic mice with expression of dominant-negative SHP-2 C459S in T cells have normal T cell development but display increased T cell activation* in vivo*, and aged mice have elevated serum antibodies [61]. In addition, phosphorylation of the adaptor LAT (linker for activation of T cells) downstream of TCR stimulation is defective in SHP-2 C459S T cells, whereas SHP-2 C459S-expression does not affect the majority of TCR-induced tyrosine phosphorylation, ERK activation, and TCR-driven proliferation [61]. These results suggest that wild-type SHP-2 plays an important role in suppressing the differentiation of T cells to a Th2 phenotype. Considering that overall TCR response is unchanged in SHP-2 C459S-expressing mice, it is likely that the phenotypes observed in the SHP-2 C459S transgenic mice may not be due to direct effects of SHP-2 on TCR-mediated signaling but rather on signaling pathways downstream of other receptors. However, this conclusion was challenged by more recent studies with selective SHP-2 depletion in thymocytes [62]. Lck-Cre-mediated deletion of SHP-2 in the thymus resulted in a significant block in thymocyte differentiation/proliferation and reduced expansion of CD4^+^ T cells. Furthermore, SHP-2-depleted mature T cells showed decreased TCR signaling *in vitro*. These data supports that SHP-2 is a common signal transducer for pre-TCR and TCR in promoting T cell maturation and proliferation.

## 5. *PTPN11* Mutations in Hematologic Malignancies

Notably, germline mutations in *PTPN11* (SHP-2) are found in *∼*50% of the cases with the developmental disorder NS [15] and nearly all patients of the developmental disorder LS that shares certain features with NS [16, 17]. Moreover, somatic mutations in *PTPN11* occur in about 35% of the patients with juvenile myelomonocytic leukemia (JMML) [18, 19], a childhood myeloproliferative disorder (MPD). In addition,* PTPN11* mutations have been identified in pediatric acute leukemias, such as myelodysplastic syndrome (MDS) (10%) [18, 19], B cell acute lymphoblastic leukemia (B-ALL) (7%) [20, 63], and acute myeloid leukemia (AML) (4%) [64]. In contrast,* PTPN11* mutations occur rarely in adult patients with MDS, AML, or chronic myelomonocytic leukemia (CMML) [65–67]. The reason for this is unclear. *PTPN11* mutations found in NS and LS are clustered in the PTP domain, whereas most of the leukemia-associated *PTPN11* mutations are located in the N-SH2 domain. NS and leukemia mutations cause changes in amino acids located at the interface formed by the N-SH2 and PTP domains in the self-inhibited SHP-2 conformation, disrupting the inhibitory intramolecular interaction, leading to hyperactivation of the catalytic activity [18]. Biochemical analyses have shown that SHP-2 mutants found in leukemias are more enzymatically active than those in NS [18, 68], suggesting that low levels of SHP-2 activation result in NS, whereas higher levels of SHP-2 activity may be required for leukemogenesis. *PTPN11* mutations found in LS are located in the PTP domain, also causing changes in amino acids located at the interface formed by the N-SH2 and PTP domains and disrupting the intramolecular interaction. However, these mutations result in inactivation of the enzymatic activity due to the changes in key catalytic amino acid residues in the phosphatase active site [69].

Recent studies have begun to elucidate the pathogenesis of *PTPN11* mutation-associated diseases. Heterozygous *PTPN11* D61G and *PTPN11 Y279C* knock-in mice develop NS, and LS phenotypes, respectively, strongly suggesting a causal role of *PTPN11 D61G* and *PTPN11 Y279C* mutations in the pathogenesis of these two diseases [70, 71]. Moreover, *PTPN11^D61G/+^* mice develop moderate MPD, characterized by excessive myeloid cell expansion and hepatosplenomegaly [70]. Myeloid progenitors from these mutant mice are hypersensitive to cytokines (GM-CSF and IL-3), reminiscent of JMML, the hallmark of which is the hypersensitive pattern of myeloid progenitor colony growth in response to GM-CSF [72]. Our recent studies showed that GM-CSF and IL-3-induced ERK and AKT activation was enhanced in *PTPN11^D61G/+^* macrophages and mast cells [73]. Clearly, aberrantly enhanced cytokine signaling contributes to the excess expansion of myeloid cells in these mutant animals. More importantly, *PTPN11^D61G/+^* mutation also aberrantly activates HSCs. This mutation accelerates HSC cycling, thereby increasing the stem cell pool and elevating short-term and long-term repopulating capabilities [73]. Inducible knock-in mice expressing the leukemogenic allele *PTPN11^D61Y^* in hematopoietic cells develop fatal MPD [74]. *PTPN11^D61Y/+^* mutation decreases the HSC pool and the percentage of HSCs in G_0_ phase in the bone marrow, whereas the number of HSCs was markedly increased and larger proportion of HSCs are quiescent in the spleen [74]. These data suggests *PTPN11^D61Y/+^* mutation drives HSCs in the bone marrow out of the quiescence, and they migrate from bone marrow to the spleen. Although global *PTPN11^D61G/+^* mice similarly show the increased HSCs in the spleen, and quiescent HSCs are also decreased in the bone marrow, the overall HSC pool in *PTPN11^D61G/+^* bone marrow is increased [73]. Since the D61Y mutation is more potent than the D61G mutation in enhancing the catalytic activity of SHP-2, the difference in HSC phenotypes caused by these two mutations suggests that the pathogenic effects of *PTPN11* mutations on HSC homeostasis is dependent on the level of SHP-2 catalytic activity. In addition, stem cell microenvironments of these two mouse models may also contribute to the different HSC phenotypes. Unlike *PTPN11^D61Y/+^* inducible knock-in mice where the mutation is primarily within the hematopoietic compartment, *PTPN11^D61G/+^*global knock-in mice carry the same mutation in all tissues and cells, including endothelial, osteoblasts, and other stromal cells that compromise the microenvironment for HSCs. *PTPN11^D61G/+^* mutation in the microenvironment may exert certain detrimental effects on HSC homeostasis by altering cytokine/growth factor secretion. Thus, the HSC phenotypes caused by *PTPN11^D61G/+^* germline mutation may represent a combined effect of *PTPN11* mutation in HSCs and in the microenvironment.

The molecular mechanisms by which *PTPN11* mutations induce hematopoietic malignancies are not fully understood. Hyperactive RAS signaling is the central event in the abnormal growth of malignant myeloid cells. Somatic *RAS* point mutations are found in ~20% of JMML [75] and ~40% of CMML cases [76], and *NRAS *or *KRAS* mutations occur in ~20% of AML specimens [77]. Given the positive role of SHP-2 in the RAS pathway, it is possible that *PTPN11* mutations contribute to hematopoietic malignancies also by deregulating RAS-ERK signaling. However, other signaling pathways may also contribute to *PTPN11*-associated leukemogenesis. Studies from our laboratory and others have shown that leukemia-associated *PTPN11* mutations also enhance multiple other hematopoietic signaling cascades, such as PI3K/AKT and JAK/STAT pathways [73, 74, 78, 79]. Mutant SHP-2 also has increased interactions with Gab2, Grb2, and p85, contributing to enhanced activation of these pathways [78]. Intriguingly, catalytically inactive SHP-2 E76K with an additional C459S mutation retained the capability to increase interaction with Gab2 and to enhance activation of the PI3K pathway, suggesting that in addition to the elevated catalytic activity, fundamental changes in physical and functional interactions between gain-of-function (GOF) mutant SHP-2 and signaling partners also play an important role in cytokine hypersensitivity. This notion is further supported by the fact that both NS and LS are associated with increased risk of hematologic malignancies although *PTPN11* mutations found in NS and LS activate and inactivate SHP-2 enzymatic activity, respectively. It, thus, appears that catalytic activity of mutant SHP-2 is not fully responsible for the pathogenic effects. Rather, increased adaptor function of mutant SHP-2 also contributes to the enhanced downstream signaling. Consistent with this idea, LS mutations, like NS mutations, also enhance SHP-2 binding to signaling partners due to its protein conformational changes resulting from the mutations [69]. It would be interesting to see how LS mutation *PTPN11 Y279C* affects hematopoietic cell development in the mutation knock-in mice [71].

Other recent studies provide additional new insights into the mechanisms by which *PTPN11* mutations induce hematologic malignancies. Konieczna et al. have found that the effect of leukemia-associated SHP-2 mutants on myeloid cell transformation involves inactivation of interferon consensus sequence-binding protein (ICSBP), which is an interferon-regulatory transcription factor that functions as a leukemia tumor suppressor [80]. ICSBP tyrosine phosphorylation during myelopoiesis is required for transcription of *NF1*, which subsequently inactivates cytokine-activated RAS, thereby creating a negative feedback mechanism for cytokine-induced proliferation [81]. Consequently, dephosphorylation of ICSBP by constitutively active mutants of SHP-2 inhibits ICSBP-dependent *NF1 *transcription, thereby impairing this negative feedback mechanism on cytokine-activated RAS and contributing to the proliferative phenotype in myeloid malignancies. In addition, the same group reported that HoxA10, a homeodomain transcription factor that represses myeloid differentiation genes, is also a substrate for SHP-2. As tyrosine phosphorylation of HoxA10 decreases its DNA-binding activity, enhanced dephosphorylation of HoxA10 by constitutively active SHP-2 synergizes with HoxA10 overexpression to accelerate disease progression to AML [82].

## 6. Perspectives

 Since its discovery, tremendous progress has been made in understanding the physiological functions of SHP-2 and its clinical relevance to human diseases. However, there are still numerous questions that remain to be addressed. All of the available data support that GOF mutations in *PTPN11* play a causal role in NS, LS, and JMML; however, whether the contribution of *PTPN11 *mutations to acute leukemias represents a primary event or a second hit acquired during disease progression is not conclusive. Moreover, upregulating the RAS pathway may be necessary but not sufficient for *PTPN11* mutations to cause leukemias. Further studies are needed to find new signaling pathways and molecules disturbed by leukemia-associated mutant SHP-2. Finally, our current understanding is very limited regarding the role of SHP-2 in maintaining HSC functions. Fully characterizing how *PTPN11 *mutations deregulate HSC activity may provide new insights into the pathogenesis of *PTPN11* mutation-associated acute leukemias and improve stem cell-based therapies.
